# Ischemic Colitis in an Endurance Runner

**DOI:** 10.1155/2012/356895

**Published:** 2012-10-10

**Authors:** Chase Grames, Cristóbal S. Berry-Cabán

**Affiliations:** ^1^Department of Family Medicine, Womack Army Medical Center, Fort Bragg, NC 28310, USA; ^2^Department of Clinical Investigation, Womack Army Medical Center, Fort Bragg, NC 28310, USA

## Abstract

A 20-year-old female running the Marine Corps Marathon developed diarrhea at mile 12. After finishing the race she noted that she was covered in bloody stool. A local emergency department suspected ischemic colitis. After discharge, her primary care physician instructed her to discontinue the use of all nonsteroidal anti-inflammatory drugs. Her symptoms resolved and she returned to running without any complications. This paper describes the pathophysiology, diagnostic approach, and management options.

## 1. Introduction

Endurance competitions have become increasingly popular among the general population. From 1980 to 2008 there has been a 197% increase in the number of marathon finishers [1]. The increasing number of amateur athletes participating in endurance events provides physicians with a new set of challenges from managing chronic pain to dealing with race-day injuries. There have been more and more reports of ischemic colitis presenting in young endurance athletes. This case report examines ischemic colitis in a young female who developed bloody diarrhea during the 2010 Marine Corps Marathon.

Endurance athletes suffer a myriad of gastrointestinal symptoms during competition and training to include bloating, abdominal cramps, diarrhea, fecal incontinence, heart burn, nausea, vomiting, chest pain, urge to defecate, and bloody stools [2–4]. Gastrointestinal symptoms are reported in 20–50% of endurance athletes, more commonly in females, and more often in runners [5]. Symptoms appear to be more debilitating with longer distances or with greater intensity [6].

## 2. Case Presentation

A 20-year-old white female student running a marathon in Washington presented to the first aid station at the finish line with dark red bloody diarrhea. Within the first 10 miles she developed an urge and she had multiple small bowel movements between miles 12 and 14. She finished without further incident, but noted bloody stools. Prior to the marathon she had no gastrointestinal complaints.

She had a photorefractive keratectomy 3 weeks prior to the race for which she was prescribed ibuprofen (800 mg 3X/D) which she continued during her training period. She denied any recent tobacco, alcohol, or illicit drug use and her family history was noncontributory.

Her physical exam was normal; vital signs were stable with the exception of an increased heart rate and evidence of bloody stools on her clothing. Transported to a local hospital she was intravenously rehydrated and discharged without any further work out. The following day she consulted her primary care physician for continuing bloody diarrhea. He advised to discontinue ibuprofen and instructed a colonoscopy if symptoms persisted. Her diarrhea resolved spontaneously without further evaluation.

## 3. Discussion

### 3.1. Pathophysiology

Ischemic colitis is most commonly seen in the elderly with cardiovascular disease. In younger patients it is seen as a complication resulting from medications, hypercoagulable conditions such as systemic lupus erythematosus, or vasculitis such as polyarteritis nodosa [7].

Every segment of the colon can be affected by ischemic colitis. The most frequent location is the descending colon followed by the ascending colon. Ischemic colitis is mainly reported in the area from the left side of the transverse colon to the descending colon corresponding to the region of overlapping marginal blood supply due to the anastomosis between the superior and inferior mesenteric arteries known as Griffith's point [8].

During exercise the sympathetic nervous system is stimulated resulting in a blood flow shunt from the splanchnic system towards the muscles [2, 4–6]. This is the primary cause of ischemic colitis in athletes. It takes a 75% reduction for up to 12 hours in the mesenteric blood flow to induce microscopic changes in the healthy colon. Exercising at 70% of maximal oxygen consumption (VO_2_ max) causes a splanchnic blood flow reduction up to 60–70% and more intense exercise can reduce blood flow up to 80% [5, 6, 9, 10].

Dehydration is another factor that can be worsened by catecholamine suppression of the thirst response center [6, 11]. Hyperosmolar sport drinks and gels create a transluminal shift of water into the colon, thus contributing to the diarrhea and dehydration [6]. The splenic flexure and the rectosigmoid junction are the watershed areas in the colon that are more prone to ischemia due to their lack of sufficient blood supply [3].

Not only are endurance athletes at risk for ischemic colitis, but many are at increased risk from chronic nonsteroidal anti-inflammatory drugs (NSAID) use. NSAIDs are commonly used to enable the athlete to return to training or competition. One study reported that athletes competing in speed and power sports use more NSAIDS than the general population [11]. At the 2000 Summer Olympics in Sydney, 26% of athletes had used NSAIDs within three days prior to competition. Others studies reported 38% of athletes at the Sydney Olympics had used NSAIDs and 33% of athletes at the 1996 Summer Olympics in Atlanta. NSAID use was highest among softball players in Atlanta (60%) and gymnasts in Sydney (100%) [11–13]. With the increase in use of nonprescription medications among athletes, providers must be aware of the potential side effects [14]. While 20% of athletes have reported side effects while taking NSAIDs, 9% reported gastrointestinal symptoms such as stomach pain, heart burn, or diarrhea [11]. It is well documented that NSAID use can lead to gastritis and peptic ulcer disease in the normal population; however it is unclear if NSAID use causes gastrointestinal bleeding in endurance athletes [6, 15].

### 3.2. Diagnosis and Management

Ischemic colitis in endurance runners is characterized by frequent, loose bowel movements during or immediately after a run. “Runner's diarrhea” is most common in long-distance or marathon runners. The cause of runner's diarrhea is not clear. One theory is that extreme exercise directs blood flow away from the intestines—contributing to diarrhea.

Most laboratory tests are normal unless the ischemia is severe, resulting in leukocytosis. If that is the case, then there may be a leukocytosis, metabolic acidosis, or elevations in lactate and lactate dehydrogenase [9]. Imaging performed to rule out other etiologies of abdominal pain reveal thumbprinting, air-filled loops, and mural thickening in 21% of plain abdominal X-rays. Barium enema is reported to be abnormal in 75% of patients but is replaced by endoscopy and computed tomography (CT) [9]. CT reveals abnormalities in up to 90% of patients with ischemic colitis [9]. Even though colonoscopy is the most sensitive method for diagnosis, there are no endoscopic findings that are specific for ischemia and can often be difficult to differentiate from inflammatory bowel disease [9]. Mesenteric angiography is only recommended when there is concern for acute arterial mesenteric infarction [10].

In mild-to-moderate cases of ischemic colitis supportive care with intravenous fluids, bowel rest, and close monitoring for fever and peritoneal signs is sufficient. In more moderate-to-severe cases empiric broad spectrum antibiotics can be considered to reduce the risk of bacterial translocation and sepsis. Although there is insufficient data to support the use of antibiotics, it is generally accepted because of the difficulty in determining what cases progress to gangrenous colitis. Most patients will clinically improve within 24–48 hours, but 20% will require surgery for resection of necrotic bowel [9].

Evidence suggests that increased use of NSAIDs by endurance runners may be a contributory factor of ischemic colitis. Athletes presenting with abdominal pain and diarrhea should be assessed for these predisposing factors. Based on a clinical research study of 611 subjects, 86.1% of gastrointestinal (colonic) mucosal lesions are likely related to NSAID use [16]. Future research should encompass the challenges of diagnosis and management of individual patient symptoms.

In reviewing similar cases, physicians should consider dosage, formulation, and necessity of NSAID regimens [15, 16]. Discontinuation of NSAIDs in our case likely assisted in the “spontaneous resolution” of symptoms. The patient presented had a full recovery, enabling her to return to running three days later. She has since participated in other marathons without complications.
